# *REV3L*, a Promising Target in Regulating the Chemosensitivity of Cervical Cancer Cells

**DOI:** 10.1371/journal.pone.0120334

**Published:** 2015-03-17

**Authors:** Li Yang, Tingyan Shi, Fei Liu, Chunxia Ren, Ziliang Wang, Yingyi Li, Xiaoyu Tu, Gong Yang, Xi Cheng

**Affiliations:** 1 Department of Gynecologic Oncology, Fudan University Shanghai Cancer Center, Shanghai, China; 2 Department of Gynecologic Oncology, Zhejiang Cancer Hospital, Hangzhou, Zhejiang, China; 3 Cancer Institute, Fudan University Shanghai Cancer Center, Shanghai, China; 4 Department of Obstetrics and Gynecology, Zhongshan Hospital Fudan University, Shanghai, China; 5 Department of Oncology, Shanghai Medical College, Fudan University, Shanghai, China; 6 Department of Gynecology and Obstetrics, People's Hospital of Wuxi, Wuxi, Jiangsu, China; 7 Department of Pathology, Fudan University Shanghai Cancer Center, Shanghai, China; University of Quebec at Trois-Rivieres, CANADA

## Abstract

REV3L, the catalytic subunit of DNA Polymerase ζ (Polζ), plays a significant role in the DNA damage tolerance mechanism of translesion synthesis (TLS). The role of *REV3L* in chemosensitivity of cervical cancer needs exploration. In the present study, we evaluated the expression of the Polζ protein in paraffin-embedded tissues using immunohistochemistry and found that the expression of Polζ in cervical cancer tissues was higher than that in normal tissues. We then established some cervical cancer cell lines with *REV3L* suppression or overexpression. Depletion of *REV3L* suppresses cell proliferation and colony formation of cervical cancer cells through G_1_ arrest, and *REV3L* promotes cell proliferation and colony formation of cervical cancer cells by promoting G_1_ phase to S phase transition. The suppression of *REV3L* expression enhanced the sensitivity of cervical cancer cells to cisplatin, and the overexpression of *REV3L* conferred resistance to cisplatin as evidenced by the alteration of apoptosis rates, and significantly expression level changes of anti-apoptotic proteins B-cell lymphoma 2 (Bcl-2), myeloid cell leukemia sequence 1 (Mcl-1) and B-cell lymphoma-extra large (Bcl-xl) and proapoptotic Bcl-2-associated x protein (Bax). Our data suggest that *REV3L* plays an important role in regulating cervical cancer cellular response to cisplatin, and thus targeting *REV3L* may be a promising way to alter chemosensitivity in cervical cancer patients.

## Introduction

Cervical cancer is the fifth common and the fourth deadliest cancer in women worldwide with nearly 528,000 new cases and 266,000 deaths in 2012[1]. Chemotherapy is one of the most useful strategies in systematic treatment of cervical cancer. Cisplatin monotherapy or in combination with other chemotherapeutic drugs remained the dominant systemic therapeutic modality for locally advanced and metastatic cervical cancer for several decades. However, the development of resistance to chemotherapeutic agents poses a major impediment that contributes to tumor recurrence, progression, and certain death[2].

Although the exact underlying mechanisms are not fully understood, studies have shown that some DNA damage escapes repair and can stall the replication machinery despite the existence of DNA repair mechanisms. For example, translesion DNA synthesis (TLS) allows damaged cells to complete genome replication by recruitment of specialized DNA polymerases to stalled replication forks[3,4]. TLS polymerases contribute to the maintenance of the genomic integrity, and otherwise stalled DNA replication forks can collapse into structures and cause a DNA double strand break (DSB), thereby to increase genomic instability[3]. Meanwhile, low-fidelity DNA polymerases are involved in spontaneous and DNA damage–induced mutagenesis, thus contributing to malignant transformation[5,6,7]. The activation of TLS may also contribute to the acquired drug resistance in tumor cells treated with DNA-damaging anticancer agents, and this is because Polζ belonging to the functional group of TLS DNA polymerases plays a major role in the bypass of many types of DNA damage[8,9,10,11]. The *REV3L* gene, the mammalian ortholog of the Saccharomyces cerevisiae *Rev3* gene, encodes the catalytic subunit of Polζ[12,13], whereas REV7L (also known as MAD2L2) interacts with REV3L through a specific binding domain [14,15,16,17].

The *REV3L* gene appears to be ubiquitously expressed in both normal and malignant human tissues, while its expression level varies in different normal and tumor cells[18,19,20]. The unique function of *REV3L* is of unusual interest because of its critical role in preventing cisplatin cytotoxicity. For example, chicken DT40 cells deficient in *Rev3* showed higher sensitivity to cisplatin, compared to other DNA repair or check-point mutants[21]. *REV3* depletion also increases sensitivity and decreases mutagenesis induced by cisplatin in mouse B-cell lymphomas and lung cancer cells, human and mouse fibroblast cells, and human colon carcinoma cells[22,23,24,25]. Suppression of the expression of *REV1L*, a member of the Y-type polymerase family that supports the activity of DNA polymerase ζ[13,26], can markedly reduce the rate of development of drug resistance in human ovarian carcinoma cells[27]. In addition, one study found that inhibition of *REV3* expression per se can induce persistent DNA damage and growth arrest in cancer cells in several lung, breast, mesothelioma, and colon tumor cell lines[28]. These results suggest that the *REV3L* gene significantly affects cellular resistance to cisplatin. Therefore, it is possible to overcome cisplatin resistance through the inhibition of *REV3L*.

Our previous study showed that Polζ expression can be used as the predictor for poor prognosis, which might be caused by the potential chemoradiation resistance in cervical cancer patients[29]. The roles of Polζ in regulating the chemoradiation resistance and predicting prognosis remain poorly characterized in cervical carcinoma, which deserves further exploration. Therefore, we established cervical cancer cell lines with down-regulation or up-regulation of *REV3L* and evaluated their sensitivity to cytotoxic agent cisplatin and related apoptosis events.

## Materials and Methods

### Ethics statement

All research involving human participants were approved by Ethics Committee at Fudan University Shanghai Cancer Center (FUSCC). A written informed consent was obtained from all recruited individuals, and each clinical investigation was conducted according to the principles expressed in the Declaration of Helsinki consent.

### Tissue Samples and Cell Lines

We made tissue microarrays using squamous cell carcinoma samples from 123 consecutive cervical cancer patients with FIGO (International Federation of Gynecology and Obstetrics, 2009) stages IB, IIA or IIB and 17 patients with normal cervical treated between March 2008 and March 2009 at FUSCC. The tissues were histopathologically confirmed independently by two gynecologic pathologists (TXY and YG). The detailed clinical formation was extracted from the patients’ electronic database at FUSCC, as described previously[29].

The established human cervical cancer cell lines SiHa, HeLa, ME180 and MS751 were obtained from American Type Culture Collection (ATCC). All cells were maintained in Dulbecco’s modified Eagle’s medium (DMEM, HyClone, Thermo Scientific, USA) supplemented with 10% fetal bovine serum (Gibco, Life technologies, USA), 100 U/ml penicillin (Biowest, Nuaillé, France), and 100 U/ml streptomycin (Biowest, Nuaillé, France) and incubated at 37°C in a humidified atmosphere with 5% CO_2_.

### Immunohistochemistry Assay

Immunohistochemistry (IHC) assays were carried out as described previously[29]. The 10×12 tissue microarray (TMA) was made by FUSCC Tissue Bank, as described previously[30]. IHC was performed on 5-μm-thick TMA sections using the antibody against Polζ (sc-48814, rabbit polyclonal antibody, Santa Cruz Biotechnology, CA, USA, 1:100 dilution) and ChemMate^TM^ EnVision^TM^/HRP (horseradish peroxidase), *Rabbit/Mouse*, detection kit (DAKO, Glostrup, Denmark). A known positive case sample was included as a positive control, and the primary antibody was replaced with nonimmune mouse/rabbit serum for negative control. The IHC staining was scored independently by two gynecologic pathologists (TXY and YG) who were blinded to patient clinical outcomes, using a scoring system based on both percentage of positive tumor cells and staining intensity, as described previously[31]. Finally, the assessment of the protein expression was defined as negative (≤2+) and positive (>2+to <6+), and for scores that were uninterpretable because of tissue loss or lack of tumor cells, a score of not applicable (N/A) was assigned.

### Plasmid Construction and Cell Transfection

All of the primers used in the study were designed using Primer Premier 5 software. To test for possible repetitive sequences, primers were aligned with the GeneBank database using the BLAST online tool. AutoDimer Software was used in the detection of potential hairpin structures and possible primer-dimer combinations. All primers were synthesized by Sangon Biotech Co., Ltd. (Shanghai, China). Three short hairpin interfering RNA (shRNA) targeting *REV3L* were designed and chemically synthesized and inserted in pBABE/U6/Puro vector according to the previously reported method[32,33]. We selected one shRNA with a highest inhibition efficiency using the cell lines with high expression of *REV3L* (Forward primer: 5'-GGAGAATAGAACTATGG TGCAAGCCTACGTAGCGTCTGCACCATAGTTCTATTCT CCCTTTTTG-3'; Reverse primer: 5'-AATTCAAA AAGGGAGAATAGAACTATGGTG CAGACGCTACGTAGGCTTGCACCATAG TTCTATTCTCC-3'). The pBABE/U6/Puro vector containing negative control (NC) shRNA (shGFP) was similarly constructed by directly inserting oligo nucleotides encoding small hairpin RNA against green fluorescence protein mRNA (shGFP) into pBabe/U6/puromycin[32,34]. Retroviruses expressing *REV3L* shRNA or GFP shRNA were produced by transfection of pBabe/U6/sh*REV3L* or pBabe/U6/shGFP into phoenix amphotropic cells and used to infect target cells by using a method described before[32]. In short, cells were infected with virus supernatants, and after a 24-hour recovery, the cells were selected with puromycin (200 ng/mL) for 10–14 days to establish stable cell lines expressing shREV3L or shGFP. The resulting cells were grown in the medium without puromycin and used for further experiments. Cell lines with low expression of *REV3L* were transfected with the pcDNA3.1/neo-REV3L plasmid (provided by Dr. Yoshiki Murakumo, Nagoya University Graduate School of Medicine, Nagoya, Japan) or the pcDNA3.1/neo negative control plasmid using Lipofectamine 2000 per the manufacturer’s instructions. After 48 h and within 120 h of transfection, cells were used for further analysis.

### RT-PCR and Real-Time PCR

Total RNA was isolated by using Trizol reagent (Invitrogen, Life technologies, USA) following the manufacturer’s protocol, and reversely transcribed into cDNA using PrimeScript^TM^ RT reagent Kit (Takara Biotechnology, Shiga, Japan). PCR products were amplified with TaKaRa Taq TM with reactions of 30 cycles of (94°C, 30 s; 58°C, 30 s; and 72°C, 1 min) using the Mastercycler® eprealplex (eppendorf AG, Hamburg, Germany). Five microliters of PCR products was analyzed by electrophoresis on 1.5% agarose gel containing ethidium bromide and visualized under UV illumination. Real-time PCR was carried out in the Applied Biosystems Prism 7900 system (Applied Biosystems, Life technologies, USA) using ExScipt Syber green QPCR kit (Takara Biotechnology, Shiga, Japan) in the following conditions: an initial denaturation of 95°C for 30 s, one cycle; 95°C for 5 s; 55°C for 30 s; and 72°C for 30 s, 40 cycles; followed by a melting curve analysis to check the specificity of amplification. Each sample was tested in triplicate, and primers to Glyceraldehyde 3-phosphatedehydrogenase (GAPDH) were used in parallel reactions as internal control. Three independent experiments were done for final analyses using the 2^-ΔΔCT^ relative quantification method. The primer pairs of *REV3L* used were 5'-CGCGTCAGTTGGGACTTAAG-3' (forward primer) and 5'-ACTATCGCCAACCTCAATGC-3' (reverse primer). The primer pairs of *GAPDH* were 5'-GGCCTCCAAGGAGTAAGACC-3'(forward primer) and 5'-CAAGGGGTCTACATGGCAAC-3' (reverse primer). The primer pairs of *REV7L* were 5'-CTGGAGAAGAATGATGTGGAG-3'(forward primer) and 5'-GTGGAGGCTGGGTGATCTCA-3' (reverse primer).

### Cell Proliferation Assay and Cell Viability Assay

To evaluate cell proliferation rate, we plated 1x10^3^ cells per well in 96-well plates with 100μl maintenance medium. Cell Counting Kit-8 (CCK-8) (Dojindo Laboratories, Kumamoto, Japan) was used to monitor cell growth at 0–7 day and the number of viable cells was assessed by measurement of absorbance at 450 nm by a Microplate Reader (BioTek Instruments, Winooski, VT, USA). The proliferation index was calculated as experimental OD value/control OD value. Cell viability was also evaluated by CCK-8. We plated 5x10^3^ cervical cancer cells per well in 96-well plates. The next day, the cells were treated with various concentrations of anticancer drugs. Cell viability was then measured as described above. Three independent experiments were done in quadruplicate wells.

### Colony Formation Assay

Cells were plated at a low density of 500 in a six-well plate in triplicate. The cells were allowed to grow for 2 weeks before being fixed with ice-cold methanol and stained with Crystal violet. The experiments were done at least three times for final analyses.

### Western Blotting

Cells were lysed in lysis buffer containing protease inhibitors (150 mM NaCl, 50 mM Tris–HCl, pH 8.0, 0.05 M EDTA, 1% Triton X-100, 0.1%SDS and 0.005×protease inhibitor cocktail). The cell lysates were resolved on 10%–15%SDS-PAGE electrophoresis and transferred to PVDF membranes (Merck Millipore, Darmstadt, Germany). The membrane was blocked with 5% nonfat milk in PBS-Tween 20 for 1 h at room temperature and incubated with selected primary antibody with β-actin used as an internal control. Immunoreactivity was detected after incubation with a horseradish peroxidase–conjugated secondary antibody (Jackson Immuno Research Laboratories, Inc., PA, USA, 1:2000 dilution) by using the enhanced chemiluminescence method (Thermo Scientific, USA). Antibodies against Bcl-2, B-cell lymphoma-extra large (Bcl-xl), Myeloid cell leukemia-1 (Mcl-1), Bcl-2-associated x protein (Bax), cytochrome c, β-actin (Santa Cruz Biotechnology, CA, USA, 1:1000 dilution) and cleaved caspase 3 p17-specific Antibody (Proteintech, IL, USA, 1:1000 dilution) were used for Western blotting analysis. Western blot band density were analysed using the Image J software per the manufacturer’s instruction, and the band density ratio of each protein to β-actin were calculated accordingly. Three independent experiments were done for final analyses.

### Cell Cycle Analysis by Flow Cytometry

Flow cytometry cell cycle analysis was performed using the PI single staining method. Cells were suspended in 1 mL phosphate buffered solution (PBS) and fixed in centrifuge tubes in 3 mL of absolute ethanol. The cells were kept in ethanol overnight at -20°C. Then, the ethanol-suspended cells were centrifuged for 5 min at 2000 rpm. The cell pellet was resuspended in 5 mL of PBS for approximately 30 s and centrifuged at 2000 rpm for 5 min. The cell pellet was resuspended in 500 μL of PI staining solutionand kept in the dark at 37°C for 10 min. The sample was analyzed using a FACScan flow cytometry (BD Biosciences, San Jose, CA, USA).

### Apoptosis Analysis by Flow Cytometry

Adherent cells were collected and subjected to the following apoptosis assays. Cells were labeled with FITC-conjugated Annexin V and PI using an Annexin V-FITC (fluorescein isothiocyanate) apoptosis detection kit (BD Biosciences, San Jose, CA, USA) according to the manufacturer’s instructions, and subsequently analyzed by FACScan flow cytometry.

### Immunofluorescence assay

Cells were plated at a density of 2000 in 24-well chamber slides and treated with different concentrations of cisplatin for 24 hours. The cells were fixed with MeOH and permeabilized with Triton X-100. Then cells were blocked with 1%BSA in PBS for 2 hours, and incubated with primary antibody against γ-H_2_AX (Biolegend, San Diego, CA, USA, 1:200 dilution) overnight. Finally, the cells were incubated with AlexaFluor-488 goat-anti-mouse secondary antibody (Jackson Immuno Research Laboratories, Inc., PA, USA, 1:200 dilution) and mounted with DAPI. γ-H_2_AX foci were assessed with green fluroscence. At least three independent experiments were conducted.

### Cisplatin treatment

Cisplatin was purchased from Sigma-Aldrich(St. Louis, MO,USA). Stock concentration of cisplatin was 5mg/ml. Different concentrations of cisplatin were used to treat cervical cancer lines for cell viability assay for different time. And in apoptosis analysis, cisplatin were used by the 30%, 50% inhibitory concentration calculated from the cell viability assay.

### Statistical Analysis

All values were expressed as means ± standard error of the mean (SEM). Statistical analysis was conducted by Student’s *t*-test. A *P* value less than 0.05 was considered statistically significant. All statistical analyses were performed using SPSS version 19.0 (SPSS Inc., Chicago, IL, USA).

## Results

### Polζ is significantly highly expressed in cervical cancer tissues

**Fig 1:**
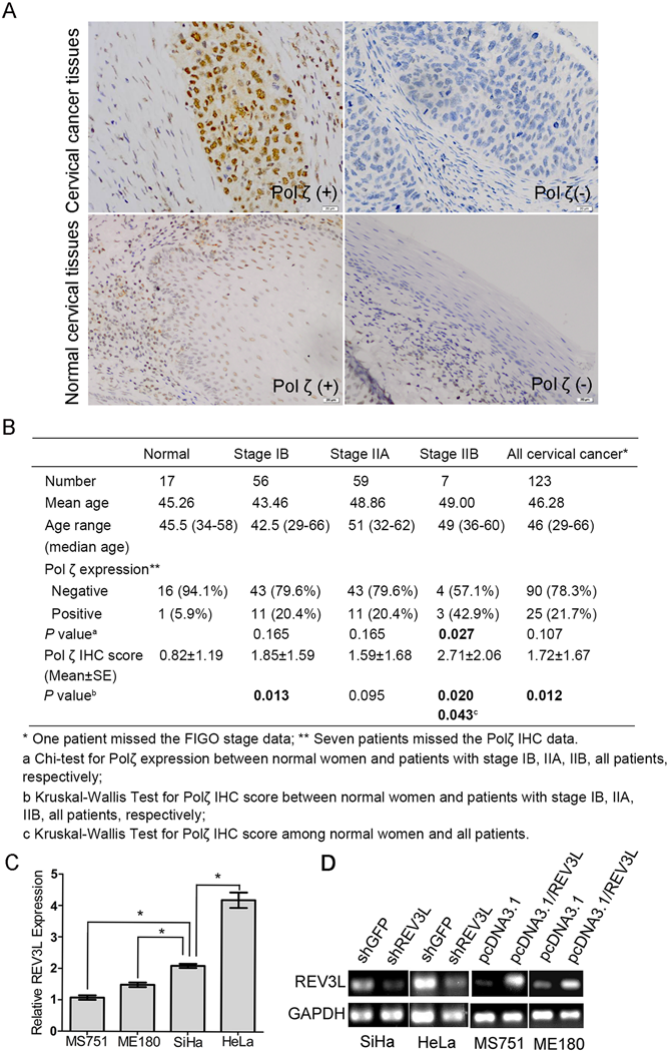
Polζ expression in cervical cancer specimens and normal cervical tissues and mRNA expression of *REV3L* in cervical cancer cell lines. (A) Polζ-positive (upper, left panel) and Polζ-negative (upper, right panel) expression in cervical cancer specimens, Polζ-positive (lower, left panel) and Polζ-negative (lower, right panel) expression in normal cervical tissues. (B) The clinical characteristics and the status of Polζ expression of cervical cancer patients and patients with nomal cervix. The mean Polζ expression was higher in the cervical cancer tissues than that in the normal cervical tissues. (C)Real time PCR analysis for *REV3L* mRNA expression in four cervical cancer cell lines. *REV3L* expression was higher in HeLa and SiHa cells than in MS751 and ME180 cells. (D) Reverse transcription PCR analysis for *REV3L* mRNA expression in short hairpin RNA transfected negative control cells (shGFP), shREV3L cells, *REV3L*-overexpressing cells, and vector control cells. *REV3L* expression was significantly decreased in shREV3L cells and was increased in the *REV3L*-overexpressing cells compared with control cells.

To investigate the difference in the expression of *REV3L* between human cervical cancer and normal cervix, the tissue microarray of 123 squamous cell carcinoma of cervical cancer patients and 17 patients with normal cervix was obtained and Polζ expression was analyzed by using IHC assay (Fig. 1A). The patients’ characteristics and the status of Polζ expression were shown in Fig. 1B. Among the 123 cervical cancer patients, seven (6%) tissues were scored of N/A for the IHC staining. In general, Polζ positive expression was detected in 21.7% (25/115) in the cervical cancer tissues and 5.9%(1/17) in the normal cervical tissues. Also, the mean Polζ expression was higher in the cervical cancer tissues than that in the normal cervical tissues (The IHC staining score was 1.72 and 0.82, respectively; *P* = 0.034) (Fig. 1A, B).

### Establishment of cervical cancer cell lines with *REV3L* knockdown or overexpression

To study the function of *REV3L* in human cervical cancer cells, we detected the *REV3L* mRNA expression in several cervical cancer cell lines. And then we set up cell line models genetically manipulated for *REV3L* expression. As shown in Fig. 1C, *REV3L* expression was higher in cervical cancer cell lines HeLa and SiHa than ME180 and MS751. Thus, we suppressed *REV3L* expression in HeLa and SiHa cell lines by stable shREV3L transfection (Fig. 1D). In contrast, *REV3L* expression was overexpressed in cervical cancer cell lines ME180 and MS751 compared with the vector control cells (Fig. 1D). Thus, we enhanced the expression of *REV3L* in ME180 and MS751 cells. Regulation of *REV3L* gene expression in established cell lines did not have a significant effect on REV7L mRNA expression levels (S1 Fig).

### *REV3L* controls cell proliferation and cell cycle

**Fig 2:**
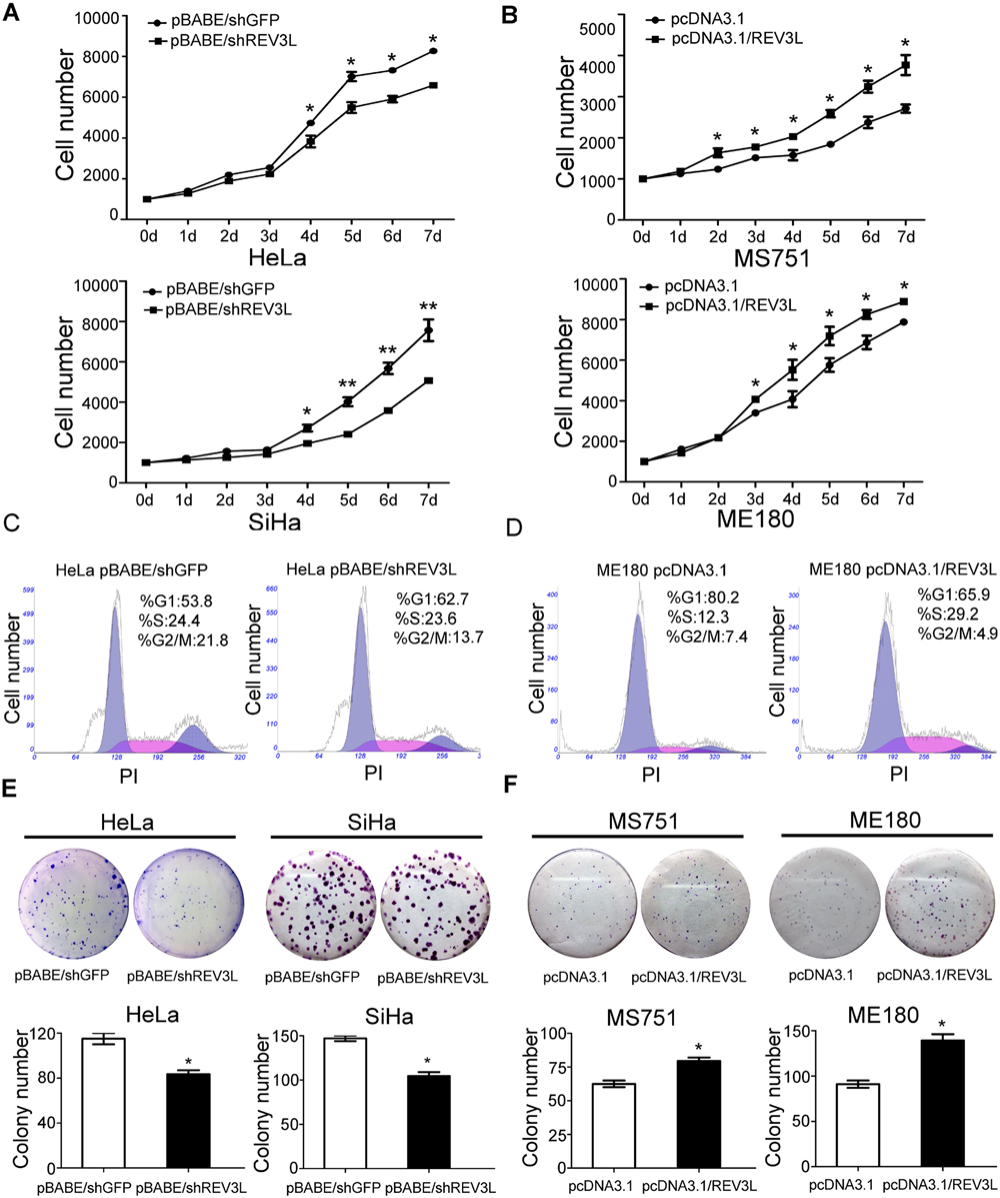
Influence of upregulation or downregulation of *REV3L* on cell proliferation, cell cycle, and colony formation ability. (A) Depletion of *REV3L* suppressed cell proliferation of HeLa shREV3L cells and SiHa shREV3L cells. (B) Enhancement of *REV3L* promoted cell proliferation of MS751 *REV3L* and ME180 *REV3L* cells. (C) Depletion of *REV3L* induced G_1_ arrest in HeLa shREV3L cells. (D) Enhancement of *REV3L* promoted G_1_ phase to S phase transition in ME180 *REV3L* cells. (E) Depletion of *REV3L* suppressed colony formation of HeLa shREV3L and SiHa shREV3L cells. (F) Enhancement of *REV3L* promoted colony formation of MS751 *REV3L* and ME180 *REV3L* cells. Data are means of three independent experiments ± SEM. * *P*< 0.05.

We first detected the effect of *REV3L* on cell proliferation and cell cycle and found that *REV3L* depletion suppressed cell growth as examined by the CCK8 assay (Fig. 2A) and colony formation assay compared with control cells (Fig. 2E). The fraction of HeLa cells in the G_1_ phase increased to 63% after inhibition of *REV3L* expression compared with 54% in control cells (Fig. 2C). Thus, inhibition of *REV3L* induces a G_1_ arrest in cervical cancer cells. Overexpression of *REV3L* promoted cell proliferation (Fig. 2B) and colony formation of cervical cancer cells (Fig. 2F). Cell cycle analysis showed that a significant decrease in the fraction of G_1_ phase was observed in ME180 cells after *REV3L* overexpression (80%) in comparison with control cells (66%) (Fig. 2D). Thus, overexpression of *REV3L* promotes G_1_ phase to S phase transition in cervical cancer cells.

### *REV3L-*depletion sensitizes cervical cancer cell lines to cisplatin

**Fig 3:**
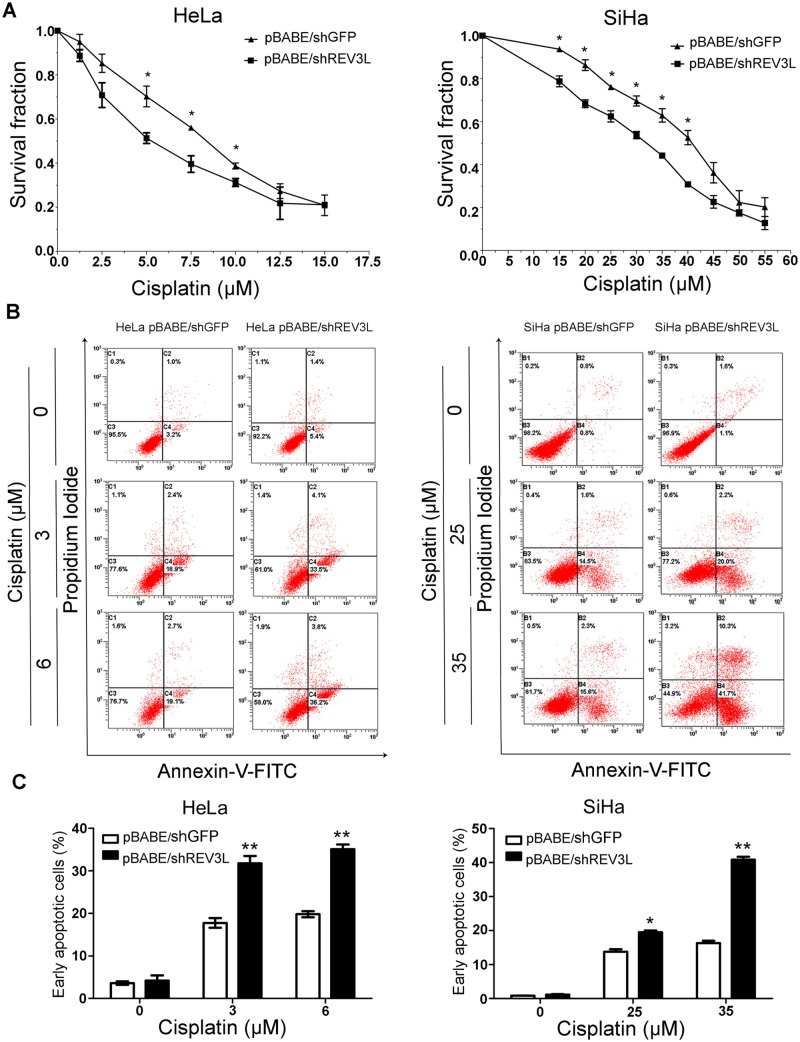
*REV3L* knockdown sensitizes cervical cancer cells to cisplatin. (A) Suppression of *REV3L* confered cervical cancer cells more sensitive to the cytotoxic effect of cisplatin. (B) Suppression of *REV3L* showed hypersensitivity to cisplatin. Cells were treated with the indicated concentrations of cisplatin for 24 h, followed by staining with Annexin V–fluorescein isothiocyanate (FITC) and propidiumiodide (PI) for early apoptotic cells (Annexin V+ PI–). Early apoptotic rates of the *REV3L*-suppression cells were significantly higher than those of the vector control cells under the same condition. (C) The percentage of apoptotic cells induced by cisplatin. Data are means of three independent experiments ± SEM. * *P*< 0.05, **P< 0.01.

*REV3L* RNAi was used to suppress the expression of *REV3L* to see whether inhibition of *REV3L* expression could enhance the chemosensitivity of human cervical cancer cells to cisplatin. The results from CCK8 assays showed that after suppression of *REV3L*, cervical cancer cells were more sensitive to the cytotoxic effect of cisplatin (Fig. 3A). Cell apoptosis was analyzed by using Annexin V-FITC staining. The data showed that early apoptotic rates in shREV3L HeLa and SiHa cells were significantly higher than in shGFP HeLa and shGFP SiHa cells in response to different doses of cisplatin for 24 h (Fig. 3B, C). The 30%, 50%, 70% inhibitory concentrations of cisplatin in HeLa, SiHa *REV3L* suppression cells and control cells are shown in Table 1.

**Table 1:** Different inhibitory concentrations (IC) of cisplatin in HeLa, SiHa shREV3L cells and control cells.

	HeLa pBABE/shGFP	HeLa pBABE/shREV3L	SiHa pBABE/shGFP	SiHa pBABE/shREV3L
IC30	5.57±0.81	2.87±0.04	31.90±0.67	28.50±1.80*
IC50	8.34±0.57	5.42±0.05*	39.90±0.10	35.80±0.97*
IC70	12.57±0.12	10.20±0.03*	49.95±1.29	45.04±0.46*

IC30, 30% inhibitory concentration of cisplatin; IC50, 50% inhibitory concentration of cisplatin; IC70, 70% inhibitory concentration of cisplatin.

*P<0.05.

### *REV3L* overexpression confers resistance to cisplatin in cervical cancer cell lines

**Fig 4:**
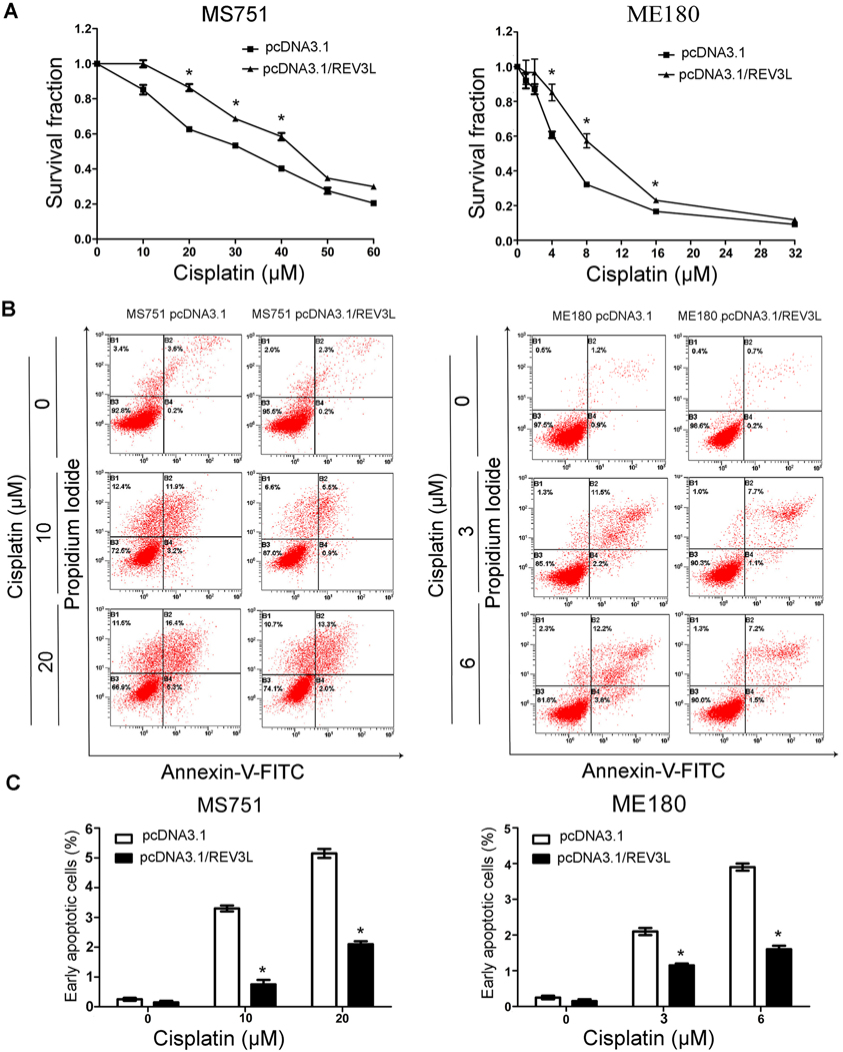
*REV3L* overexpression rendered resistance to cisplatin in cervical cancer cells. (A) Overexpression of *REV3L* rendered cervical cancer cells resistant to cisplatin. (B) Detection of apoptotic cells by flow cytometry. Overexpression of *REV3L* inhibited apoptosis induced by cisplatin. (C) The percentage of apoptotic cells induced by cisplatin. Data are means of three independent experiments ± SEM. * *P*< 0.05.

To further validate the effect of *REV3L* on the chemosensitivity of human cervical cancer cells to chemotherapeutic drugs, we examined the sensitivity of *REV3L* overexpression cells to cisplatin. The results showed that overexpression of *REV3L* rendered the cells resistant to various doses of cisplatin (Fig. 4A). The *REV3L*-overexpressing ME180 and MS751 cells showed a decrease in cisplatin-induced early apoptosis (Fig. 4B, C). The 30%, 50%, 70% inhibitory concentrations of cisplatin in MS751, ME180 *REV3L* overexpression cells and control cells are shown in Table 2.

**Table 2:** Different inhibitory concentrations (IC) of cisplatin in MS751, ME180 *REV3L* cells and control cells.

	MS751 pcDNA3.1	MS751 pcDNA3.1/REV3L	ME180 pcDNA3.1	ME180 pcDNA3.1/REV3L
IC30	21.83±2.35	31.73±1.80	3.33±0.30	6.43±0.90*
IC50	33.47±2.10	43.50±1.70*	5.93±0.30	10.10±0.90*
IC70	51.50±2.25	59.67±1.30*	10.57±0.30	16.10±0.65*

IC30, 30% inhibitory concentration of cisplatin; IC50, 50% inhibitory concentration of cisplatin; IC70, 70% inhibitory concentration of cisplatin.

*P<0.05.

### *REV3L* induced chemoresistance is mediated through the mitochondria-associated apoptotic pathway

**Fig 5:**
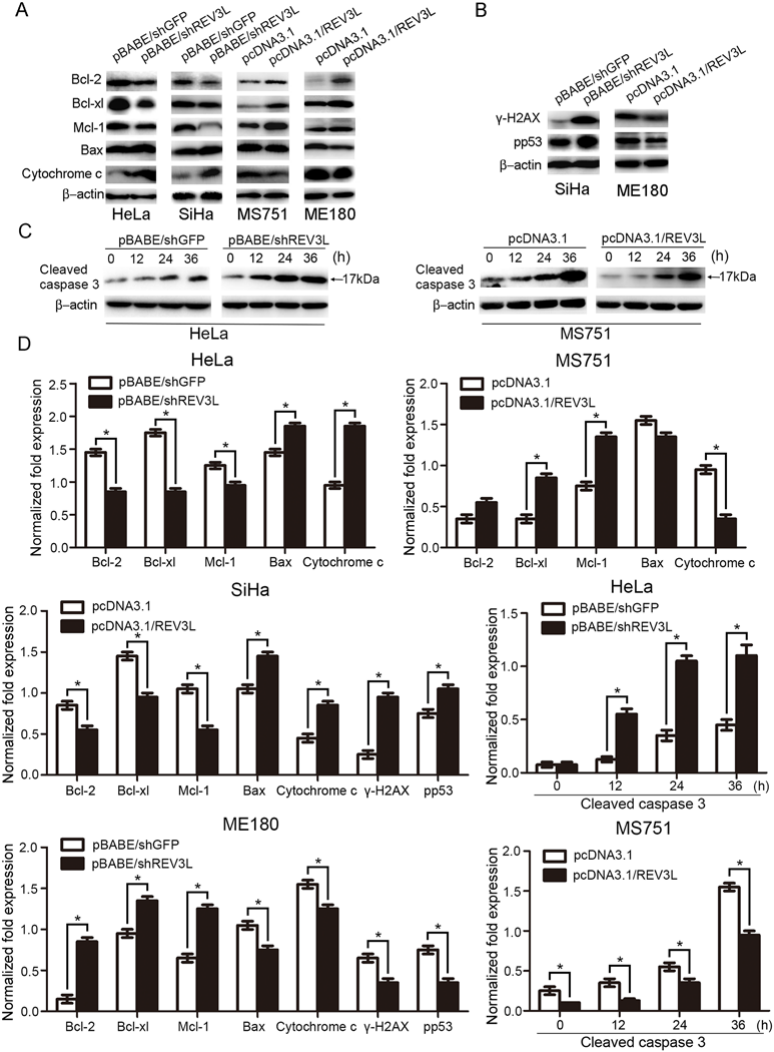
Alteration of mitochondria-associated apoptotic proteins after cisplatin treatment. (A)The expression levels of Bcl-2, Bcl-xl and Mcl-1 were lower and levels of Bax and cytochrome c were higher in shREV3L cells compared with shGFP cells in response to the same dose of cisplatin. *REV3L*-overexpressing MS751 and ME180 cells showed higher levels of Bcl-2, Mcl-1 and Bcl-xl and lower levels of Bax and cytochrome c compared to the vector control cells. (B) γ-H_2_AX and p-p53(pS15) proteins were increased in SiHa shREV3L cells and decreased in ME180 REV3L cells, compared with control cells after cisplatin treatment. (C) Time-dependent expression of cleaved caspase-3 after exposure to a single dose of cisplatin (1 μmol/L) in MS751 cells and a single dose of cisplatin (10 μmol/L) in HeLa cells. Expression levels of cleaved caspase-3 were lower in MS751 *REV3L* cells and higher in HeLa shREV3L cells compared with control cells in response to the same dose of cisplatin in a time-dependent manner. (D) Normalized fold expression of each protein against internal control protein. Data are means of three independent experiments ± SEM. * P< 0.05.

As the mitochondria-associated apoptotic pathway participates in cellular response in tumors to many anticancer drugs, to see whether it is involved in *REV3L* induced alteration in sensitivity to cisplatin, we performed an analysis of proapoptotic proteins (Bax, cytochrome c) and antiapoptotic proteins (Bcl-2, Bcl-xl, and Mcl-1) in the vector control cells and the *REV3L*-overexpressing or suppression cells with cisplatin treatment (Fig. 5A, D). After treatment with cisplatin for 24 h, Bcl-2, Bcl-xl and Mcl-1 levels markedly decreased, and Bax and cytochrome c levels increased in shREV3L HeLa and SiHa cells, compared with shGFP HeLa and SiHa cells. Consistently, after treatment with cisplatin, *REV3L*-overexpressing MS751 and ME180 cells showed higher levels of Bcl-2, Mcl-1 and Bcl-xl and lower levels of Bax and cytochrome c, compared to the vector control cells. Furthermore, after exposure to cisplatin (1 μmol/L) for 72 h, a time dependent increase in the amounts of cleaved caspase-3 was observed in HeLa shREV3L cells and a decrease in the amounts of cleaved caspase-3 was observed in MS751 *REV3L* cells after a single dose treatment of 10 μmol/L of cisplatin (Fig. 5C, D). Thus, these results suggest that *REV3L* confers chemoresistance to cisplatin through the mitochondria- associated apoptotic pathway.

### *REV3L* limits the DNA damage response after treatment with cisplatin

**Fig 6:**
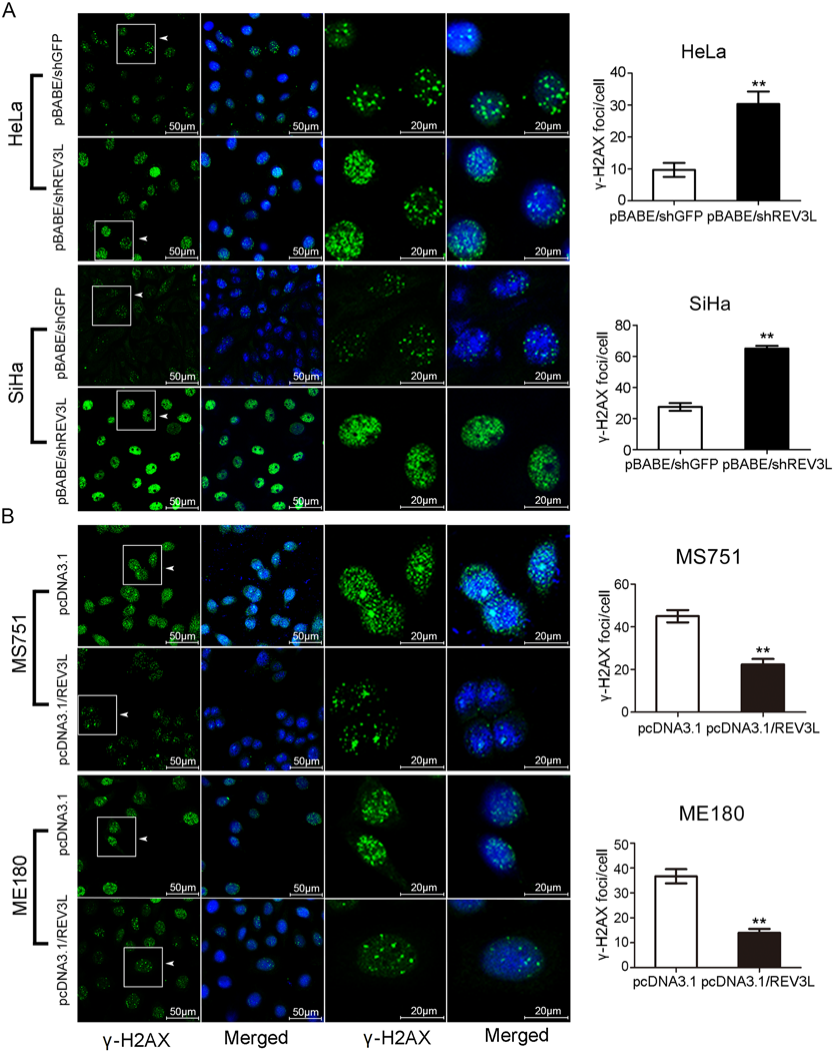
Variation of γ-H2AX foci formation in cervical cancer cell lines after upregulation or downregulation of *REV3L* with cisplatin treatment. (A) HeLa and SiHa cells were cultured in the presence of in a single dose of cisplatin (3 μmol/L and 25 μmol/L, respectively) for 24 h. HeLa and SiHa cells deficient in *REV3L* expression exhibited intenser γ-H_2_AX staining in comparison with shGFP control cells after exposure to cisplatin. (B) MS751 and ME180 cells were cultured in the presence of in a single dose of cisplatin (30 μmol/L and 5 μmol/L, respectively) for 24 h. MS751 and ME180 cells with *REV3L* overexpression showed weaker γ-H_2_AX staining than control cells. Quantification of γ-H_2_AX foci was performed and analysed per cell. Over 50 cells were counted in each cell line.

To determine the intensity of DNA damage response after cisplatin treatment in *REV3L* overexpression or suppression cervical cancer cell lines, we detected γ-H_2_AX (pS139) foci formation using immunofluorescence. HeLa and SiHa cells deficient in *REV3L* expression exhibited intense γ-H_2_AX staining in comparison with shGFP control cells after exposure to cisplatin (Fig. 6A). On the contrary, MS751 and ME180 cells with *REV3L* overexpression showed weaker γ-H_2_AX staining compared with control cells (Fig. 6B). Analysis of proteins showed that γ-H_2_AX, and p-p53(pS15) proteins were increased in SiHa shREV3L cells and decreased in ME180 *REV3L* overexpression cells, compared with control cells after cisplatin treatment (Fig. 5B, D).

## Discussion

Polζ is an error-prone DNA polymerase involved in TLS that is characterized by its ability to extend mismatched primer-template termini. On one hand, Polζ can maintain the genome stability and is benefit for survival when cells are exposed to DNA damage[35,36,37,38,39]. On the other hand, through inducing spontaneous and DNA-lesion–triggered mutations by causing incorrect nucleotides in DNA, Polζ contributes to the accumulation of genetic damage, and thus may play a role in carcinogenesis and tumor progression[40,41,42]. Expression levels of the *REV3L* gene, which encodes the catalytic subunit of Polζ, vary in different types of cancer. Some studies found that *REV3L* was overexpressed in human gliomas tissues resected before therapy compared with normal brain tissues[19], mismatch repair-defective, p53-/- colorectal adenocarcinomas compared with control tissues[43]. *REV3L* polymorphisms were also reported to be significantly associated with risk of lung cancer and breast cancer[44,45]. Whereas other studies found that the *REV3L* expression was downregulated in colon carcinomas independent of tumor grade[46], gastric cancer[47], and lung cancer[47]. In this study, we found that the expression levels of Polζ were significantly higher in cervical cancer tissues than that in normal cervix using IHC, Polζ may promote tumor formation in cervical cancer. However, further studies are needed to elucidate the role of Polζ in carcinogenesis and tumor progression.

The effects of *REV3L* depletion on cancer cell growth have been controversial. For example, previous studies showed that inhibition of *REV3L* expression in HCT116, U2OS, and HeLa cancer cells did not alter cell growth/survival[18,48]. Conversely, another study revealed that inhibition of *REV3L* reduced colony formation of lung, breast, mesolioma, and colon tumor cell lines[28]. *REV3L* was found to be necessary for proliferation of mouse embryonic fibroblasts and could inhibit mice lymphomas formation[49]. In our study, depletion of *REV3L* suppressed cell proliferation and colony formation of cervical cancer cells, the overexpression of *REV3L* promoted cell proliferation and colony formation of cervical cancer cells. Inhibition of *REV3L* induced G_1_ arrest of cervical cancer cells, and overexpression of *REV3L* promoted G_1_ to S phase transition of cervical cancer cells.

Chemotherapy has been widely used in the treatment of a variety of cancers, including cervical cancer. Platinum is still the mainstay agent in the chemotherapeutic regime for cervical cancer patients, and cisplatin is one of the most commonly used. Cisplatin could induce growth arrest and apoptosis of cancer cells by forming inter- and intrastrand DNA cross-links or inducing strand breaks. However, clinical use of cisplatin is limited by the frequent resistance of cancer cells. It has been postulated that tumor mutation rate is one of a few critical determinants of clinical resistance of a variety of human cancers. The activities of *REV3* and *REV1* have been linked to the drug resistance to cisplatin and cyclophosphamide in murine models of both B-cell lymphoma and lung cancer[23,25]. Using short hairpin RNA to inhibit *REV1* or *REV3* deficient in tumor cells significantly sensitized these tumors to treatment[23,25]. In some *in vitro* studies, cultured human cell lines showed that suppressing either *Rev1* or *REV3L* reduced the rate of emergence of cisplatin resistance[19,50,51]. In this study, we found that inhibition of *REV3L* increased cellular sensitivity to cisplatin with activation of the mitochondrial apoptotic pathway in cervical cancer cells, with altered Bcl-2, Bcl-xl, and Bax expression levels. Overexpression of *REV3L* conferred resistance to cisplatin and decreased cisplatin-induced apoptosis of the mitochondria-mediated apoptotic pathway.

Previously, the persistent accumulation of H2AX phosphorylation after cisplatin treatment and exposure to high-dose ionizing radiation was observed after *REV3* depletion in cancer cells[52], suggesting the accumulation of irreparable DSBs. In this study, cells deficient in *REV3L* expression exhibited an intenser γ-H2AX staining than control cells after exposure to cisplatin, *REV3L* overexpression showed weaker γ-H_2_AX staining than control cells. Also, γ-H_2_AX, and P-P53(pS15) proteins were increased in SiHa shREV3L cells, compared with control cells after cisplatin treatment. This suggests that inhibition of *REV3L* may activate the DNA damage response to cisplatin treatment.

These observations may suggest that therapies targeting *REV3L* may hold significant promise for cancer treatment. However, genomic instability due to *REV3L* loss has been documented previously in chicken, mouse, and human hematopoietic cells[39,41,42,49,53]. Although temporary inhibition of polζ may be tolerated, caution must be used with longer-term suppression as normal tissues may experience enhanced cellular toxicity, genome instability by chromosome breakage, and additional tumor formation. Future work will be warranted to determine the function of *REV3L* in vivo, and how to achieve *REV3L* inhibition without enhancing normal cell toxicity.

## Conclusions

*REV3L* functions to confer chemoresistance to cisplatin treatment via regulation of cell cycle and apoptosis, which may be explored as a potential therapeutic target in cervical cancer treatment.

## Supporting Information

S1 Fig Expression of *REV7L* after upregulation or downregulation of *REV3L*in cervical cancer cells. Real time PCR analysis showed that suppression or enhancement of *REV3L* did not affect *REV7L* mRNA expression in cervical cancer cells.
(TIF)
