# Supplementary figures and images for: REV3L, a Promising Target in Regulating the Chemosensitivity of Cervical Cancer Cells
Source: PLoS One. 2015 Mar 17;10(3):e0120334. doi: 10.1371/journal.pone.0120334 (PMC4364373; doi:10.1371/journal.pone.0120334)

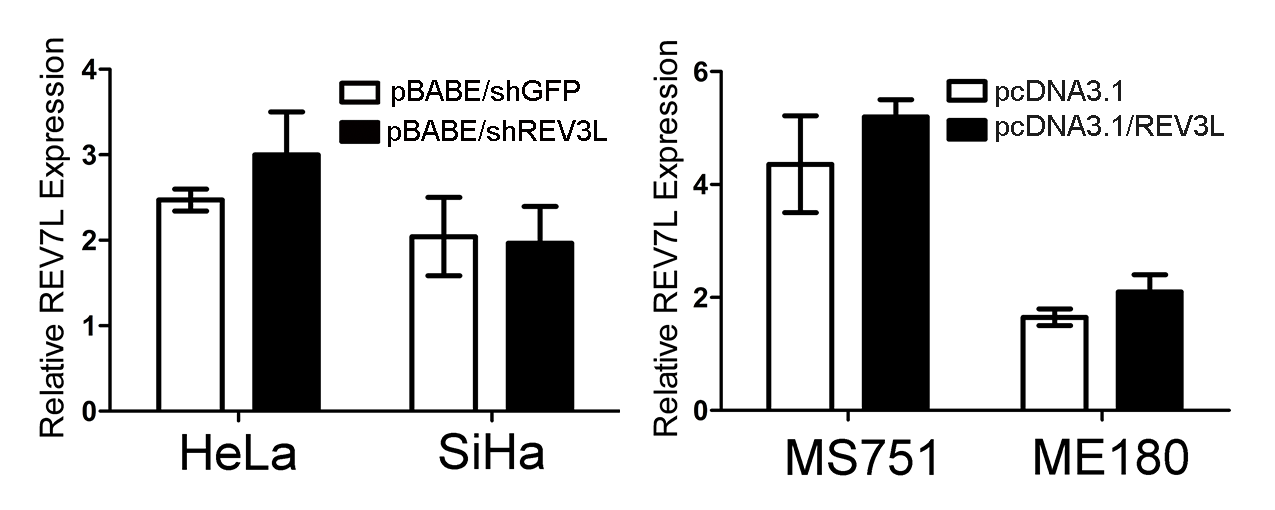

Supplement: S1 Fig — Real time PCR analysis showed that suppression or enhancement of REV3L did not affect REV7L mRNA expression in cervical cancer cells. (TIF) [file pone.0120334.s001.tif]
